# *RNF213* Mutation Associated with the Progression from Middle Cerebral Artery Steno-Occlusive Disease to Moyamoya Disease

**DOI:** 10.1007/s12975-024-01293-2

**Published:** 2024-08-27

**Authors:** Tomoki Sasagasako, Yohei Mineharu, Takeshi Funaki, Yasutaka Fushimi, Hideo Chihara, Silsu Park, Kota Nakajima, Yasuzumi Matsui, Masakazu Okawa, Takayuki Kikuchi, Yoshiki Arakawa

**Affiliations:** 1 Department of Neurosurgery, Graduate School of Medicine, Kyoto University, Kyoto, Japan; 2 Department of Diagnostic Imaging and Nuclear Medicine, Graduate School of Medicine, Kyoto University, Kyoto, Japan

**Keywords:** Atherosclerosis, Disease progression, Middle cerebral artery occlusion, Moyamoya disease, *RNF213*

## Abstract

Middle cerebral artery steno-occlusive disease (MCAD) has been recognized as a different clinical entity from moyamoya disease (MMD). Although MCAD can progress to MMD, the extent to which patients actually progress and the risk factors for this progression have not been fully elucidated. We retrospectively reviewed patients with MCAD who underwent *RNF213* genotyping. Demographic features, *RNF213* p.R4810K mutation, medical history, and longitudinal changes in angiography were analyzed. Sixty patients with 81 affected hemispheres were enrolled. During the follow-up period, 17 patients developed MMD, and the *RNF213* p.R4810K mutation was the only factor significantly associated with progression to MMD (odds ratio, 16.1; 95% CI, 2.13–731; *P* = 0.001). The log-rank test demonstrated that patients with the mutation had a higher risk of progression to MMD (*P* = 0.007), stenosis progression (*P* = 0.010), and symptomatic cerebral infarction or hemorrhage (*P* = 0.026). In Cox regression analysis the p.R4810K mutation remained a significant factor after adjusting for age group (childhood or adult onset) at diagnosis (hazard ratio, 8.42; 95% CI, 1.10–64.4). Hemisphere-based analysis also showed that the mutation was associated with a higher risk of progression to the MMD hemisphere (*P* = 0.002), stenosis progression (*P* = 0.005), and cerebral infarction or hemorrhage (*P* = 0.012). The *RNF213* p.R4810K mutation was identified as a risk factor for progression from MCAD to MMD. Genotyping for this mutation may contribute to risk stratification in MCAD.

**Supplementary Information:**

The online version contains supplementary material available at 10.1007/s12975-024-01293-2.

## Introduction

Middle cerebral artery steno-occlusive disease (MCAD) is a vascular condition that causes ischemic stroke [1]. Although atherosclerosis is recognized as the most frequent cause of MCAD, it can also be an early manifestation of moyamoya disease (MMD) [2–4]. Some family members display MCAD in familial MMD, suggesting that MCAD in these cases may represent incomplete expression or an early phase of MMD [5, 6].

MMD is characterized by progressive stenosis at the terminal portion of the internal cerebral artery (ICA) and dilated perforating arteries, forming various fragile collaterals known as moyamoya vessels. Although the involvement of the terminal portion of the ICA is a distinct characteristic of MMD, current insights have indicated that the distinctive angiographic characteristics of MMD may not be present in its initial stages [7]. In MMD, cerebral hemodynamics are impaired, causing transient ischemic attack (TIA) and cerebral infarction, whereas the dilated, fragile moyamoya vessels sometimes rupture and cause hemorrhage [8, 9]. *RNF213* p.R4810K mutation is the most common genetic risk factor for MMD in the East Asian populations [10, 11]. A recent study demonstrated that the mutation was also associated with quasi-MMD [12].

The Asymptomatic Moyamoya Registry study (AMORE study) designated a hemisphere with stenosis in the proximal portion of the middle cerebral artery (MCA) or anterior cerebral artery (ACA), whereas the ICA terminal portion remains intact, as a “questionable (Q)” hemisphere [13]. Compared with Q hemispheres, definitive moyamoya hemispheres are at a higher risk for cerebrovascular events. Moreover, revascularization surgery is more effective in patients with MMD than in those with other steno-occlusive diseases [14]. Therefore, it is beneficial to identify patients with early manifestations of MMD among those with MCAD. However, few studies have addressed the risk factors for MCAD development. We hypothesized that the p.R4810K mutation is associated with progression from MCAD (“Q” hemisphere) to definitive MMD. To test this hypothesis, we aimed to extensively investigate the risk factors for the development of MMD, including MMD and quasi-MMD, in patients with MCAD. This study will contribute to improving the management of patients with MCAD through risk stratification and to a better understanding of the progression patterns of MMD from MCAD.

## Materials and Methods

### Patient Population and Imaging Study Assessment

We retrospectively reviewed consecutive patients with intracranial artery stenosis at our institution, all of whom had been genotyped for the p.R4810K mutation in *RNF213*. The patients were initially diagnosed between December 2000 and December 2023.

We reviewed the clinical data and radiological examinations from the patient’s medical records, including 1.5- or 3.0-T magnetic resonance (MR) images and conventional angiography, if available. Radiological evaluation included the development of MMD, stenosis progression, and grade 2 periventricular anastomosis.

This study enrolled patients with MCAD who, at initial presentation, had stenosis in the M1 segment of the MCA, without any stenosis in the terminal portion of the ICA [15]. Stenosis in the terminal portion of the ICA was rigorously evaluated, adhering to established diagnostic criteria [16]. Patients presenting with a unilateral MCAD hemisphere and a contralateral moyamoya hemisphere were excluded from the study. For the included patients, each hemisphere was further categorized as “Q” or unaffected. According to the AMORE study, a “Q” hemisphere is defined as one exhibiting stenosis in the proximal portion of the MCA or ACA but not in the terminal of the ICA [13].

Two experienced neurosurgeons (TS and TF) independently evaluated the radiological examinations. The κ statistic was calculated between the two readers to assess the inter-rater reliability regarding the diagnosis of MMD development from MCAD [17]. Furthermore, a judgment committee comprising three neurosurgeons (TS, TF, and YM) reviewed and discussed the radiological examinations to confirm the final diagnoses, adhering to the official diagnostic criteria of the Research Committee on the Spontaneous Occlusion of the Circle of Willis from the Ministry of Health and Welfare, Japan [16]. In the evaluation of stenosis, the cerebral artery was categorized into nine predefined segments (basilar artery, right and left intracranial ICA, right and left A1–A2 segment of the ACA, right and left M1–M2 segment of the MCA, and right and left posterior cerebral arteries) [18]. Arterial stenosis was classified based on the validated grading scale as follows: grade 1, normal or mild stenosis (0–29% diameter stenosis); grade 2, moderate stenosis (30–69% diameter stenosis); grade 3, severe stenosis (70–99% diameter stenosis); and grade 4, occlusion (100% diameter stenosis) [19]. Progression was defined by an increase in stenosis degree by at least one grade or the development of de novo stenosis in a new segment [20].

The definition and classification of periventricular anastomosis were based on previous studies [21, 22]. Grade 2 periventricular anastomosis was characterized by a clear connection in the periventricular region between the perforating or choroidal arteries and the medullary or insular arteries. Anastomoses were classified into three subtypes: (1) lenticulostriate, beginning at the lenticulostriate artery; (2) choroidal, beginning at the anterior or posterior choroidal artery; and (3) thalamic, beginning at the thalamotuberal, thalamogeniculate, or thalamoperforating artery. In this study, unless specifically stated otherwise, “periventricular anastomosis” was classified as grade 2. For patients undergoing revascularization surgery, images acquired only before surgery were used for assessment because the surgery itself can potentially reduce antegrade blood flow and moyamoya vessels [23–26]. Patients who had undergone revascularization surgery before referral to our department were excluded.

### Clinical Variables

We measured the following clinical variables as potential confounders: age at initial diagnosis, sex, antiplatelet use, family history of MMD, medical history (hypertension, diabetes mellitus, autoimmune diseases, and other associated diseases), smoking habits (current or past smoker vs. nonsmoker), drinking habits (everyday alcohol drinker vs. occasional or nondrinker), and first sign at onset (ischemic stroke, including TIA, intracranial hemorrhage, or asymptomatic) for all participants. Patients aged < 17 years were considered to have childhood-onset MMD. MMD, a disease of unknown etiology, requires differentiation from similar cerebrovascular lesions associated with underlying autoimmune diseases, referred to as quasi-MMD. However, in this study, to elucidate the role of *RNF213* p.R4810K mutation in patients with MCAD, we included patients with underlying diseases that cause quasi-MMD. Outcome variables in this study were defined as follows: (1) development of radiological MMD, (2) stenosis progression, (3) incidence of symptomatic ischemic infarction or hemorrhage during the follow-up period, (4) revascularization surgery, and (5) detection of grade 2 PVAs at final imaging. Neither TIA nor perioperative events were included as outcomes. In patient-based analyses, if either hemisphere reaches a clinical outcome, that patient is considered to have experienced the outcome. This prevents the redundant counting of the same outcome for one patient.

### Genotyping

Genomic DNA was obtained from peripheral blood samples using a DNA Blood Mini Kit (Qiagen). Genotyping of the *RNF213* p.R4810K mutation was performed using TaqMan SNP Genotyping Assays (Applied Biosystems), as previously described [12].

### Statistical Analyses

All statistical analyses were performed using the EZR software [27]. Log-rank tests were performed to estimate the cumulative rates and the risk of progression to MMD, stenosis progression, and development of symptomatic ischemic infarction or hemorrhage during the follow-up period. In patient-based analyses, Cox and multivariate logistic regressions were performed to adjust for covariates, such as age at diagnosis. Continuous variables were compared using the Mann–Whitney *U* test, and categorical variables were analyzed using Fisher’s exact test, as appropriate. Continuous data are expressed as medians (interquartile ranges [IQRs]). *P* values < 0.05 were considered statistically significant.

## Results

### Patient Demographics

**Fig. 1:**
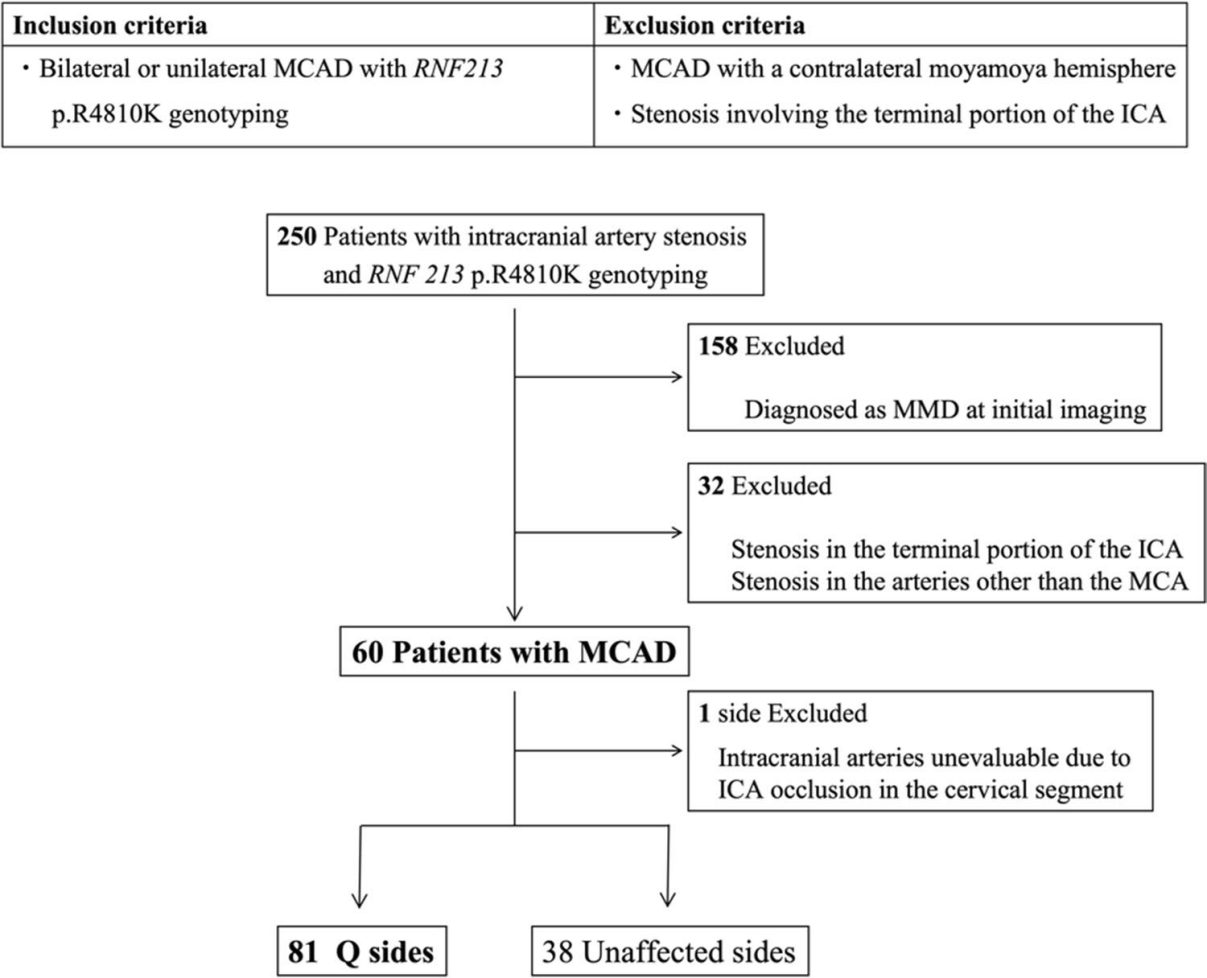
Selection process for patients with MCAD. MCAD, middle cerebral artery steno-occlusive disease

A flow diagram of the patient inclusion process is shown in Fig. 1. In total, 250 patients with intracranial artery stenosis underwent *RNF213* genotyping. Of these, 158 patients diagnosed with MMD were excluded from this study. Additionally, 32 patients were excluded because of stenosis in arteries other than the MCA or stenosis in the terminal portion of the ICA that was not diagnosed as MMD because moyamoya vessels were not observed. This resulted in 60 patients with MCAD being included in this study, comprising 81 with Q hemispheres and 38 with unaffected hemispheres. Among the Q hemispheres, 78 had stenosis or occlusion in the M1 segment, whereas three hemispheres exhibited isolated stenosis in the A1 segment with contralateral M1 involvement. One hemisphere was excluded from the evaluation because the intracranial arteries could not be assessed owing to occlusion of the ICA in the cervical segment. No patients underwent revascularization surgery before being referred to our hospital.

### Clinical Outcomes of Patients with MCAD

Among the 60 patients with MCAD, 17 (28.3%) exhibited progression to MMD during a median follow-up period of 74 (IQR, 38–152) months. Concerning inter-rater agreement on MMD development, the κ statistic was 0.879 (95% confidence interval [CI], 0.746–1.00), demonstrating excellent reliability. Sixteen of the 17 patients who developed MMD (94.1%) had the *RNF213* p.R4810K mutation, showing that the mutation was significantly associated with the progression from MCAD to MMD (odds ratio [OR] = 16.1; 95% confidence interval [CI], 2.13–731;* P* = 0.001) (Table 1). Of these patients, 38 (63.3%) were female, and the median follow-up duration was 74 (IQR, 38–152) months. Patients with the mutation tended to be younger at diagnosis than those with wild-type alleles, although this association was not statistically significant (*P* = 0.053). Thus, logistic regression analysis was performed to adjust for age at diagnosis, and the p.R4810K mutation was independently associated with the risk of progression to MMD after that adjustment (OR, 14.3; 95%CI, 1.72–120; *P* = 0.014).

**Table 1:** Clinical characteristics of patients with MCAD

	**Q → Q**	**Q → MMD**	***P* value**
Number of patients	43	17	-
Median (IQR) age at initial diagnosis, y	44 (31–53)	32 (26–40)	0.053
Childhood-onset (< 17y), *n* (%)	5 (11.6)	4 (23.6)	0.260
Female, *n* (%)	25 (58.1)	13 (76.5)	0.241
Family history, *n* (%)	9 (20.9)	8 (47.1)	0.059
Hypertension	9 (20.9)	5 (29.4)	0.511
Diabetes	3 (7.0)	1 (5.9)	1
Hyperlipidemia	8 (18.6)	3 (17.6)	1
Autoimmune disease, *n* (%)	4 (9.3)	1 (5.9)	1
Smoking habits, *n* (%)	12 (27.9)	4 (23.5)	1
Drinking every day, *n* (%)	6 (14.0)	3 (17.6)	0.704
Symptom at onset	-	-	0.061
Ischemic stroke, including TIA, *n* (%)	24 (55.8)	15 (88.2)	-
Hemorrhage, *n* (%)	4 (7.0)	0 (0)	-
Asymptomatic, *n* (%)	15 (34.8)	2 (11.8)	-
p.R4810K mutation, *n* (%)	21 (48.8)	16 (94.1)	**0.001**
p.R4810K genotypes, *n* (%)	-	-	**0.001**
AA (homozygote)	1	0	-
GA (heterozygote)	20	16	**-**
GG (wild-type)	22	1	**-**

*The bold values indicate P<0.05; MCAD*, middle cerebral artery steno-occlusive disease; *IQR*, interquartile range; *y*, years; *IA*, transient ischemic attack; *NA*, not applicable

**Fig. 2:**
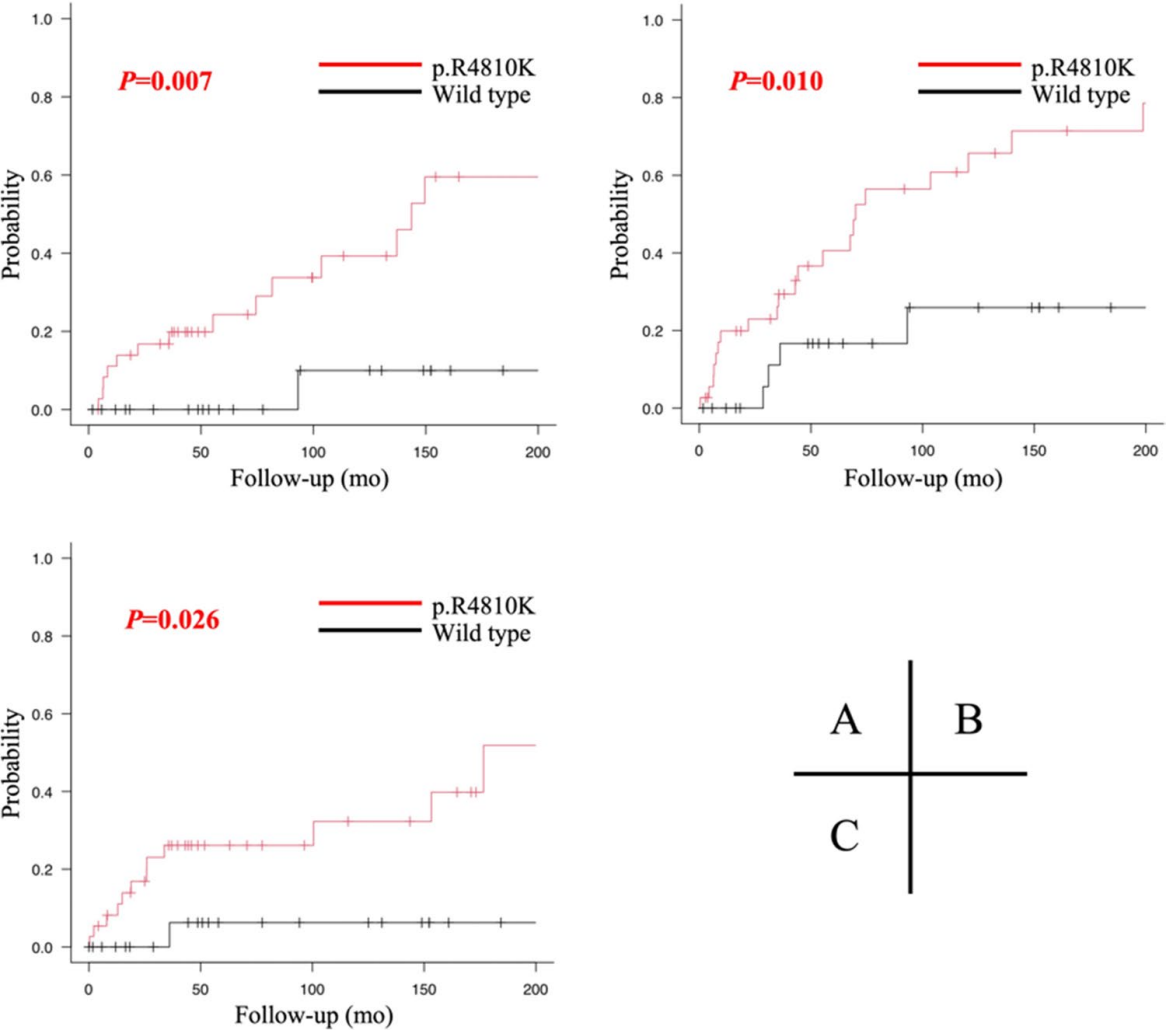
Patient-based analyses of cumulative incidence: progression from MCAD to MMD, angiographic stenosis progression, and stroke associated with the p.R4810K mutation. Cumulative incidence for (A) the development of moyamoya disease, (B) progression of stenosis, and (C) cerebral infarction or hemorrhage during the follow-up period were compared between patients with the *RNF213* p.R4810K mutation and those without it

**Fig. 3:**
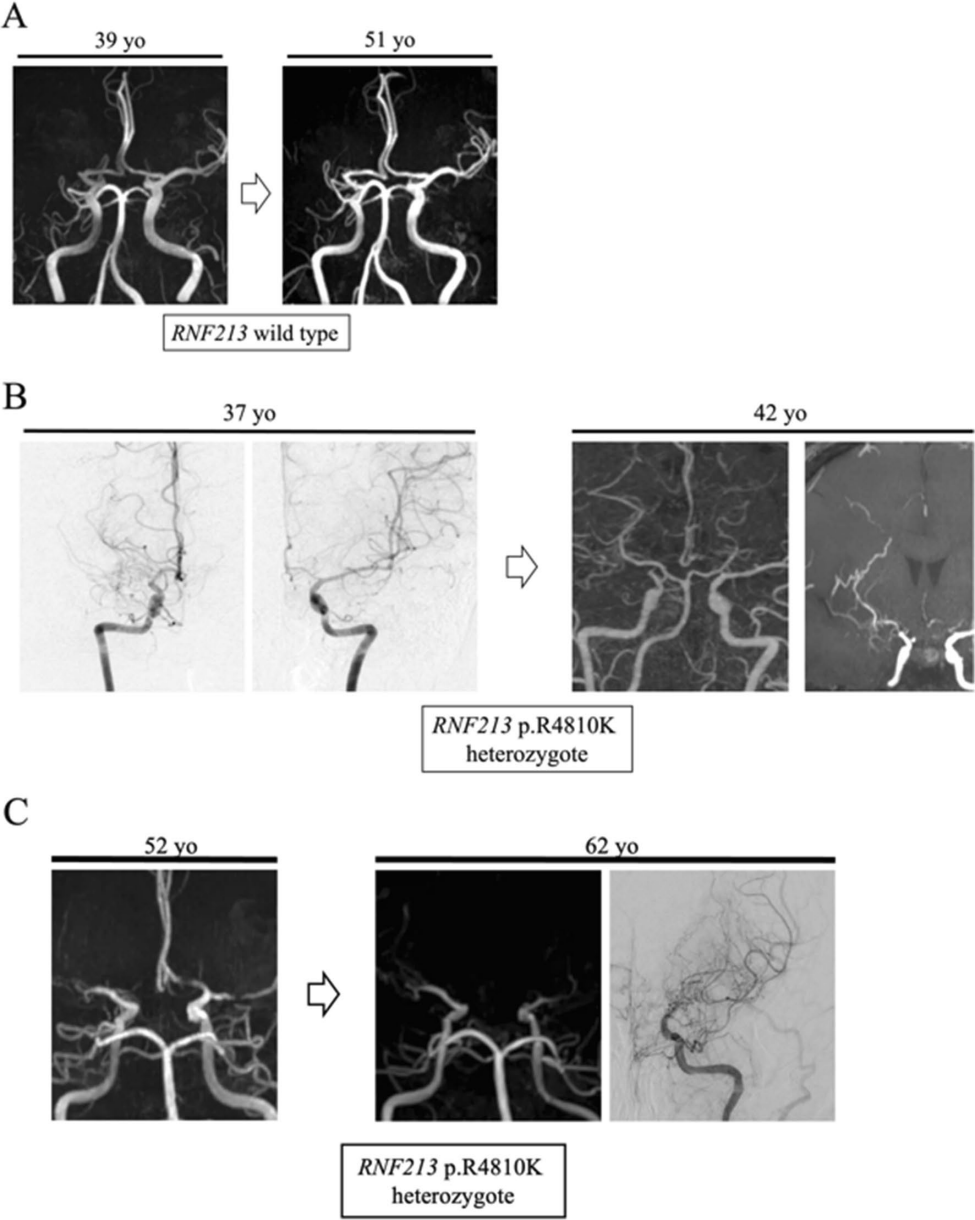
Illustrative cases demonstrating the effect of *RNF213* p.R4810K mutation on progression from MCAD to MMD. **A** A 39-year-old man who do not have the *RNF213* p.R4810K mutation presented with cerebral infarction and right MCA occlusion. No further cerebrovascular events occurred after the initiation of antiplatelet therapy. The MR angiography findings remained unchanged throughout a follow-up period of 12 years. **B** A 37-year-old woman carrying the heterozygous *RNF213* p.R4810K mutation experienced a transient ischemic attack and was diagnosed with right MCA occlusion. Over a 5-year follow-up period, MCA stenosis progressed to moyamoya disease. Despite medication, the patient experienced frequent transient ischemic attacks and subsequently underwent STA-MCA anastomosis. **C** A 52-year-old man carrying the heterozygous *RNF213* p.R4810K mutation experienced left cerebral infarction and was diagnosed with bilateral MCA stenosis. Over a 10-year follow-up period, left MCA stenosis progressed to moyamoya disease, and a recurrent cerebral infarction ultimately led to bypass surgery

The median follow-up periods were 68 (IQR, 37–140) months for the *RNF213* wild-type group and 78 (IQR, 38–161) months for the mutation group (*P* = 0.506). In the mutation group, 36 patients were heterozygous, whereas one was homozygous for the mutation. Patients with the p.R4810K mutation had a significantly higher likelihood of stenosis progression (*P* = 0.002), required revascularization surgery (*P* = 0.008), and experienced cerebral infarction or hemorrhage (*P* = 0.011). In addition, the log-rank test indicated a significant difference in the development of MMD (*P* = 0.007), progression of stenosis (*P* = 0.010), and occurrence of cerebral infarction or hemorrhage (*P* = 0.026) during the follow-up (Fig. 2A–C). To determine whether the p.R4810K mutation was a significant risk factor after adjusting for childhood- or adult-onset, Cox proportional hazard analysis was performed after adjustment for age group at diagnosis. The p.R4810K mutation remained a significant predictor of progression to MMD, with a hazard ratio of 8.42 (95% CI, 1.10–64.4; *P* = 0.040, Table S1). In stratification analysis, in the adult-onset group (*n* = 51) there was a significant association between the mutation and disease progression to MMD (*P* = 0.021). Figure 3 presents three illustrative cases of patients with the p.R4810K heterozygous mutation and those with the wild-type alleles, in terms of disease progression to MMD.

We further compared patient characteristics and clinical outcomes between patients with the p.R4810K mutation (*n* = 37) and those with the wild-type alleles (*n* = 23, Table 2). Patients with the p.R4810K mutation were diagnosed at a median age of 37 (IQR, 26–47) years, which was significantly lower than those with the wild-type alleles, who had a median age of 47 (IQR, 30–69) years (*P* = 0.044).

**Table 2:** Genotype–phenotype correlation of *RNF213* p.R4810K in patients with MCAD

MCAD patients, *n* = 60	p.R4810K wild *n* = 23	p.R4810K mutant *n* = 37	*P* value
Median (IQR) age at diagnosis, y	47 (30–69)	37 (26–47)	**0.044**
Childhood onset (< 17 y), *n* (%)	2 (8.7)	7 (18.9)	0.459
Female, *n* (%)	14 (60.7)	24 (64.9)	0.788
Family history	3 (13.6)	14 (38.2)	**0.048**
Autoimmune disease, *n* (%)	3 (13.0)	2 (5.4)	0.362
Antiplatelet use, *n* (%)	19 (82.6)	27 (73.0)	0.534
Median (IQR) follow-up, mo	68 (37–140)	78 (38–161)	0.506
Symptom at initial diagnosis	-	-	0.831
Ischemic stroke, including TIA, *n* (%)	15 (65.2)	25 (67.6)	-
Hemorrhage, *n* (%)	1 (4.3)	3 (8.1)	-
Asymptomatic, *n* (%)	7 (30.4)	9 (24.3)	-
PCA involvement, *n* (%)	0 (0)	3 (8.1)	0.279
Bilateral Q hemisphere, *n* (%)	5 (21.7)	16 (43.2)	0.104
Development of MMD, *n* (%)	1 (4.3)	16 (43.2)	**0.001**
Progression of stenosis, *n* (%)	4 (18.1)	22 (59.4)	**0.002**
Patients with revascularization surgery, *n* (%)	2 (8.7)	16 (43.2)	**0.008**
Patients with cerebral infarction or hemorrhage during follow-up period, *n* (%)	1 (4.3)	12 (32.4)	**0.011**

*The bold values indicate P<0.05; MCAD*, middle cerebral artery steno-occlusive disease; *IQR*, interquartile range; *y*, years; *mo*, month; *TIA*, transient ischemic attack; *Q*, questionable; *MMD*, moyamoya disease; *PCA*, posterior cerebral artery

### Hemisphere-Based Analysis of Clinical Outcomes in Q and Unaffected Hemispheres

Of the 81 Q hemispheres, 34% (18/53) carrying the p.R4810K mutation progressed to moyamoya hemispheres, compared to only 3.6% (1/28) of those with wild-type alleles (*P* = 0.002, Table 3). Hemispheres carrying the mutation demonstrated stenosis progression (*P* = 0.002). Among Q hemispheres with the variant, the most frequent progression patterns were de novo stenosis in the ICA (26.4%), followed by increased stenosis severity in baseline MCA lesions (15.1%) (Table S2). Additionally, hemispheres with the mutation underwent revascularization surgery more frequently than those with wild-type alleles (*P* = 0.007; Table 3). Furthermore, grade 2 periventricular anastomoses were observed in 13 (24.5%) hemispheres with the p.R4810K mutation, which was significantly higher than that in one hemisphere (3.6%) with the *RNF213* wild-type alleles (*P* = 0.028). Log-rank test also showed a significant difference in the development of MMD (*P* = 0.002), progression of stenosis (*P* = 0.005), and cerebral infarction or hemorrhage in each hemisphere (*P* = 0.012) between those with the wild-type alleles and those with the p.R4810K mutation (Fig. S1A–C).

**Table 3:** Genotype–phenotype correlation of the *RNF213* p.R4810K mutation in Q hemispheres

Q hemisphere, *n* = 81	p.R4810K wild *n* = 28	p.R4810K mutant *n* = 53	*P* value
Median (IQR) follow-up, mo	63 (26–150)	96 (37–161)	0.304
Symptom at initial diagnosis	-	-	0.926
Ischemic stroke, including TIA, *n* (%)	14 (50.0)	28 (52.8)	-
Hemorrhage, *n* (%)	1 (3.6)	3 (5.7)	-
Asymptomatic, *n* (%)	13 (46.4)	22 (41.5)	-
Progression to moyamoya hemisphere, *n* (%)	1 (3.6)	18 (34.0)	**0.002**
Progression of stenosis, *n* (%)	3 (10.7)	24 (45.2)	**0.002**
Hemispheres with revascularization surgery, *n* (%)	2 (7.1)	19 (35.8)	**0.007**
Hemispheres with cerebral infarction or hemorrhage during follow-up period, *n* (%)	0 (0)	12 (22.6)	**0.006**
**Angiographic findings at final imaging**			
Hemispheres with grade 2 PVA, *n* (%)	1 (3.6)	13 (24.5)	**0.028**
Total number of LSA type PVAs, *n* (%)	0 (0)	8 (15.1)	**0.046**
Total number of ThalA type PVAs, *n* (%)	0 (0)	1 (1.8)	1
Total number of ChoA type PVAs, *n* (%)	1 (3.6)	7 (13.2)	0.252

*The bold values indicate P<0.05; Q*, questionable; *mo*, month; *TIA*, transient ischemic attack; *PVA*, periventricular anastomosis; *LSA*, lateral striate artery; *ThalA*, thalamic artery; *ChoA*, choroidal artery

Among the unaffected hemispheres, 21 out of 38 hemispheres harbored the p.R4810K mutation. In the p.R4810K mutation group, 2 (9.5%) hemispheres developed MMD, and 6 (28.6%) hemispheres exhibited progression of stenosis. In contrast, in the *RNF213* wild-type group, no hemispheres developed MMD, and 1 (5.9%) showed stenosis progression (Table S3). The incidence rate of stenosis progression was higher in the p.R4810K mutation group than in the *RNF213* wild-type alleles group, although this was not statistically significant in the log-rank test (*P* = 0.151) (Fig. S2).

## Discussion

Our study revealed that the *RNF213* p.R4810K mutation was significantly associated with MMD development in patients with MCAD (Q case). Actually, sixteen of 37 patients carrying the mutation developed MMD, whereas only 1 of 23 patients without the mutation developed it. We also showed that a substantial proportion of patients with MCAD develop MMD, although selection bias cannot be excluded. The results of the log-rank test further support an association between the p.R4810K variant and progression to MMD (*P* = 0.007). It had been reported that the p.R4810K variant is a risk factor for MCAD, but this study newly reveals its association with the progression from MCAD to MMD. Progression from MCAD to MMD had been described in case reports [3, 4], but we demonstrated that it is not an uncommon phenomenon. Recently, retrospective studies have shown that the *RNF213* p.R4810K mutation is associated with contralateral progression in unilateral MMD [28, 29]. Analyzing 93 patients with unilateral MMD, Mineharu et al. revealed that the *RNF213* p.R4810K mutation and contralateral abnormalities on the ACA or MCA were significantly correlated with the contralateral development of unilateral MMD [28]. Although the pathophysiological functions of the p.R4810K mutation remain unclear, these lines of evidence suggest that the mutation seems to be a clinically significant indicator for developing MMD in patients with cerebral artery stenosis [7, 30].

In addition to its association with the development of MMD, the *RNF213* p.R4810K mutation is also associated with stenosis progression. A recent study reviewed 52 patients with cerebral artery stenosis without MMD and found that patients carrying the *RNF213* p.R4810K mutation had a higher risk of stenosis progression [20]. Consistent with this report, our data showed that the p.R4810K mutation provided a significantly higher risk of stenosis progression in patients with MCAD compared with the *RNF213* wild type (*P* = 0.010). The most common progression pattern was de novo stenosis in the ICA, indicating that lesions in patients with MCAD typically progress proximally, ultimately leading to the development of MMD. Furthermore, the unaffected hemispheres on initial imaging exhibited progression to the moyamoya hemisphere or de novo stenosis. Among patients with the p.R4810K mutation, 2 of 21 unaffected hemispheres (9.5%) developed MMD and 6 showed de novo stenosis (28.6%) during the median follow-up period of 71 months. An observational longitudinal study involving healthy family members of patients with MMD reported that 3 of 11 individuals (27.3%) experienced de novo stenosis during a median follow-up period of 7 years [5]. This indicates that even the unaffected hemisphere possesses a relatively high risk of cerebral artery steno-occlusive lesions in patients with the *RNF213* R4810K mutation.

MCAD is a well-established vascular lesion causing ischemic stroke; thus, evaluating underlying pathophysiology would be beneficial to determine the clinical management of patients [1, 2]. Patients with low atherosclerosis burden also experience MCAD, but their pathogenesis has long been unknown [31]. However, recent studies using high-resolution MRI have shown that non-atherosclerotic MCA stenosis occurs frequently in individuals heterozygous for the *RNF213* p.R4810K mutation [30–32]. Moreover, patients with the p.R4810K mutation in our MCAD cohort were diagnosed at a younger age compared with those without the mutation. Our results are consistent with the result of a recent study demonstrating that the *RNF213* p.R4810K mutation is common in early-onset ischemic stroke with M1 or A1 stenosis [33]. The *RNF213* p.R4810K mutation is considered a risk factor for anterior circulation stenosis, especially in younger patients with a lower burden of atherosclerosis [32].

In the current study, the *RNF213* p.R4810K mutation significantly increased the risk of cerebrovascular events (symptomatic infarction or hemorrhage) in MCAD in both patient-based and hemisphere-based analyses. Revascularization surgery was required in 19 (35.8%) hemispheres carrying the mutation, compared to only 2 (7.1%) hemispheres with wild-type alleles. Consistently, in a recent retrospective study, MMD patients with the p.R4810K mutation often exhibited symptoms in both hemispheres, while those with wild-type alleles were more likely to only exhibit symptoms in one hemisphere [34]. Although different from MCAD cases, the AMORE study prospectively followed 109 patients with asymptomatic MMD and reported that symptomatic infarction or hemorrhage occurred in 4.9% (7/143) of moyamoya hemispheres compared to 0% (0/39) of Q hemispheres during a 5-year observation period. This is consistent with our data showing that the *RNF213* mutation increased the risk of progression to moyamoya hemispheres, as well as symptomatic stroke. Although the AMORE study did not investigate *RNF213* mutations, the potential association is an interesting topic for further research.

Our data demonstrate that the *RNF213* p.R4810 mutation is significantly associated with periventricular anastomosis. Grade 2 periventricular anastomoses were detected in 13 of 53 affected hemispheres in patients carrying the mutation, compared with only 1 of 28 hemispheres in patients without the mutation (*P* = 0.028). Periventricular anastomosis is classified into three subtypes—lenticulostriate, choroidal, and thalamic—and is a substantial risk factor for cerebrovascular events in MMD [35–37]. Previously, Funaki et al. demonstrated that the marked development of choroidal anastomosis was closely associated with posterior hemorrhage in the territory of the choroidal artery [35–37]. Additionally, other types of anastomoses have also frequently been considered the vessels responsible for bleeding [25, 38]. Among patients with MMD, a recent study demonstrated that the p.R4810K mutation harbored a significantly higher risk of periventricular anastomosis development than *RNF213* wild type [39].

Stenosis of the terminal portion of the ICA is an essential diagnostic characteristic of MMD. MCAD with intact terminal ICA (Q hemisphere) has a better prognosis than MMD, and MCAD may represent an etiology different from MMD. In contrast, in the current study, some patients with MCAD with the *RNF213* p.4810 K mutation exhibited progression to definitive MMD during the follow-up period. According to current insights, the distinctive angiographic characteristics of MMD may not be present in its initial stages, particularly in adults [7]. Based on imaging techniques or patient characteristics, distinguishing MCA steno-occlusive lesions as early manifestations of MMD from other vascular pathologies remains challenging. Genotyping of *RNF213* potentially contributes to the differentiation of patients with early-stage MMD from those with MCAD. In patients with the p.R4810K mutation, close clinical follow-up is required for the early detection of stenosis progression or the development of periventricular anastomosis.

This study has some limitations. First, our findings were limited by the relatively small size of our patient cohort. Therefore, subsequent studies with larger sample sizes are required to validate our results. Second, we did not systematically analyze the *RNF213* p.R4810K mutation in all patients with MCAD presenting at our institutions, potentially introducing selection bias and affecting the reported incidence rate of MMD progression. A multicenter prospective cohort study is warranted to confirm the validity of our results. Third, the prevalence of the *RNF213* p.R4810K mutation in patients with intracranial artery stenosis, including MMD, varies with ethnicity [40]. Therefore, the external validity of our findings may be influenced by the patient’s ethnic background.

## Conclusions

Our results suggest that *RNF213* p.R4810K mutation is associated with progression from MCAD (Q hemisphere) to definitive MMD and the incidence of cerebrovascular events. Genotyping *RNF213* may contribute to risk stratification in patients with MCAD and adequate management, such as close follow-up. Further studies are required to confirm the clinical significance of *RNF213* genotyping in MCAD.

## Supplementary Information

Below is the link to the electronic supplementary material.

Supplementary file1 (DOCX 1606 KB)

Supplementary file2 (DOCX 18 KB)

## Data Availability

The data supporting the findings of this study are available from the corresponding author upon reasonable request from any investigator.

## Abbreviations

ACA: Anterior cerebral artery
ICA: Internal cerebral artery
MCA: Middle cerebral artery
MCAD: Middle cerebral artery steno-occlusive disease
MMD: Moyamoya disease
Q: Questionable
