# Supplementary material for: RNF213 Mutation Associated with the Progression from Middle Cerebral Artery Steno-Occlusive Disease to Moyamoya Disease
Source: Transl Stroke Res. 2024 Aug 27;16(4):1146–55. doi: 10.1007/s12975-024-01293-2 (PMC12202527; doi:10.1007/s12975-024-01293-2)
Supplement: Supplementary file 1 — Supplementary file1 (DOCX 1606 KB) [file 12975_2024_1293_MOESM1_ESM.docx]

**Figure S1. Questionable (Q) hemisphere-based analysis of cumulative incidence: progression from MCAD to MMD, angiographic stenosis progression, and stroke associated with the p.R4810K mutation**
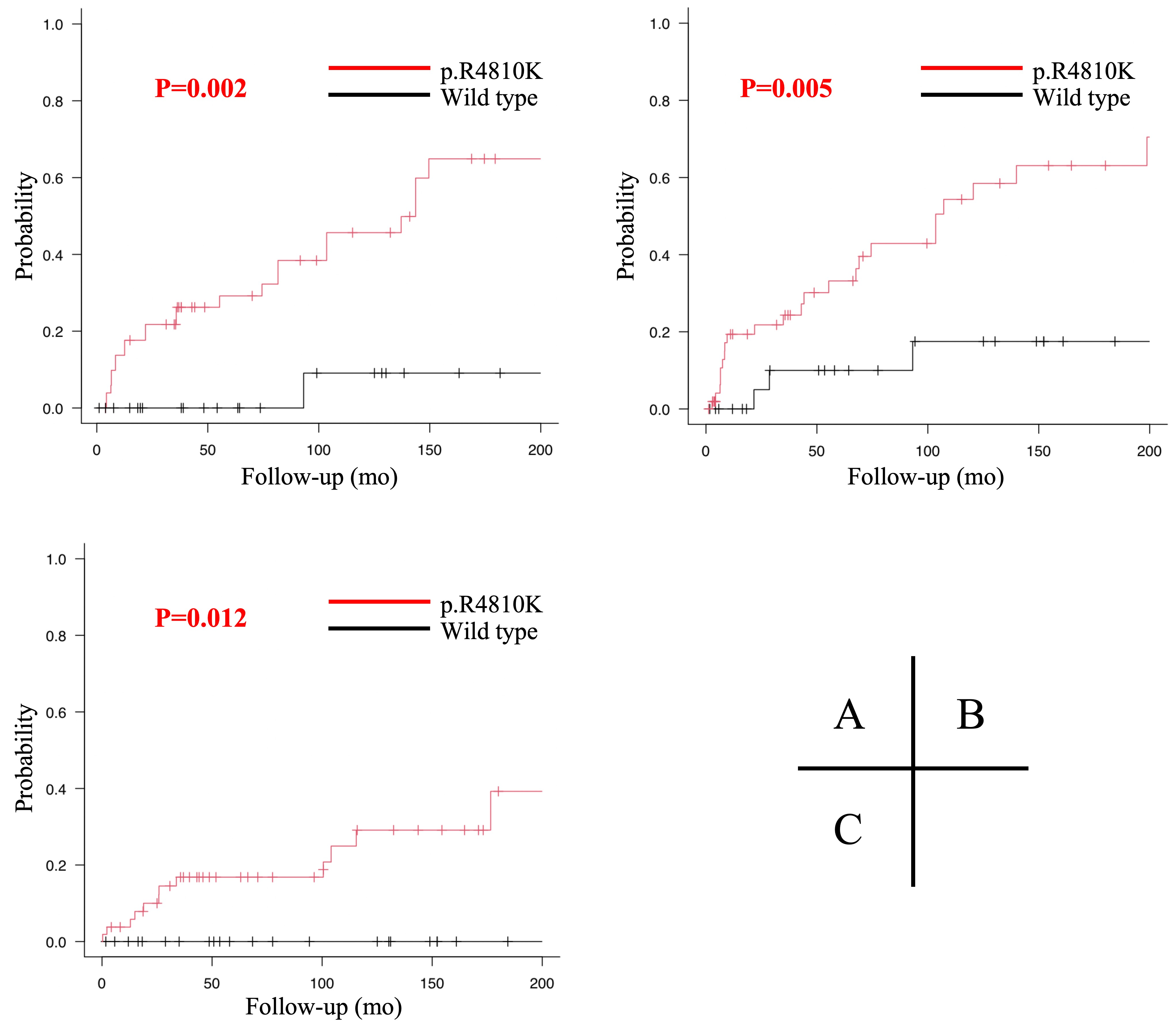


Cumulative proportion curves for (A) the development of moyamoya disease, (B) progression of stenosis, and (C) cerebral infarction or hemorrhage during the follow-up period.

**Figure S2. Cumulative incidence of stenosis progression in unaffected hemispheres with and without the p.R4810K mutation**


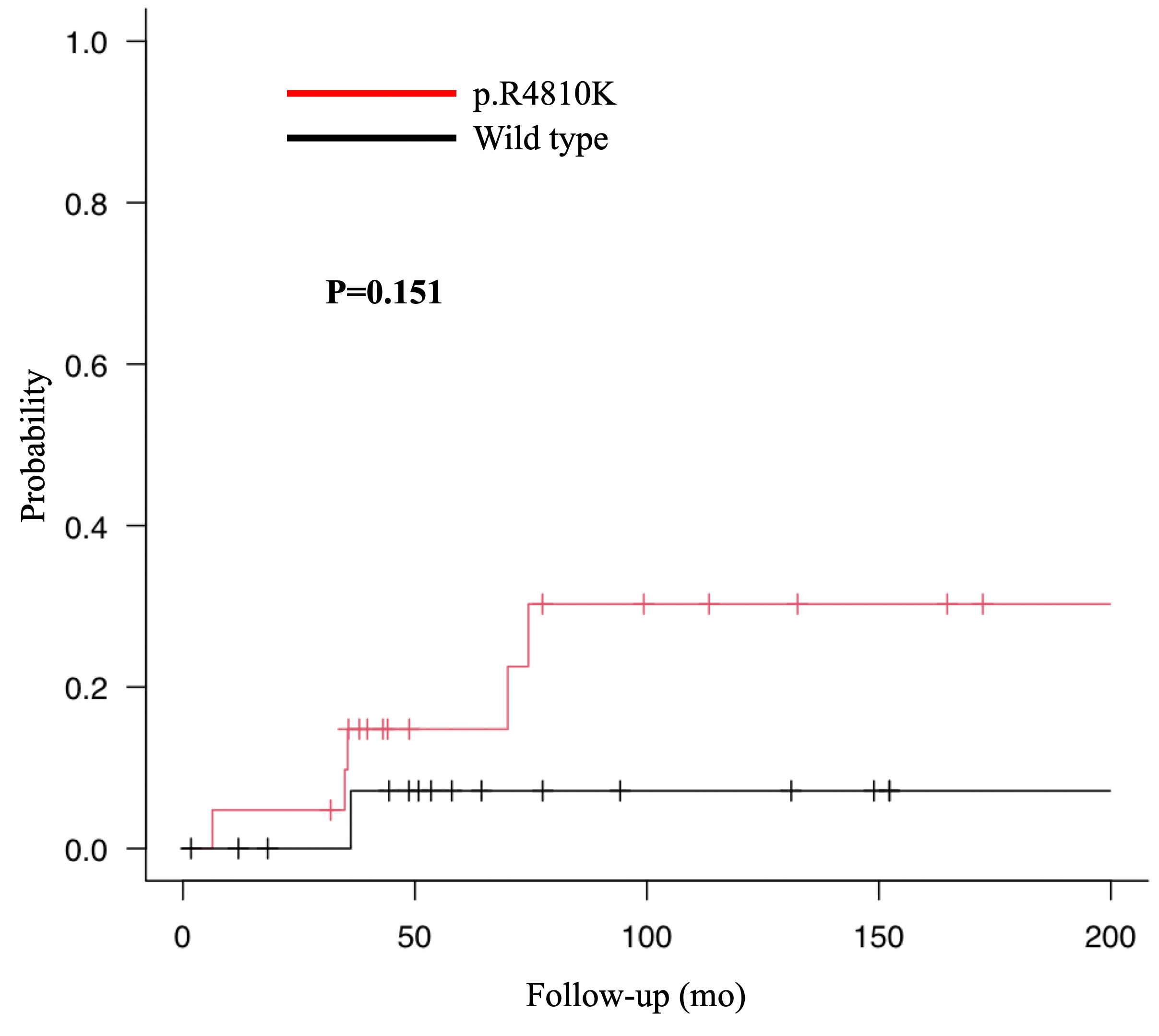


Cumulative proportion curves for the progression of stenosis in unaffected hemispheres.
