# Supplementary material for: RNF213 Mutation Associated with the Progression from Middle Cerebral Artery Steno-Occlusive Disease to Moyamoya Disease
Source: Transl Stroke Res. 2024 Aug 27;16(4):1146–55. doi: 10.1007/s12975-024-01293-2 (PMC12202527; doi:10.1007/s12975-024-01293-2)
Supplement: Supplementary file 2 — Supplementary file2 (DOCX 18 KB) [file 12975_2024_1293_MOESM2_ESM.docx]

**Table S1. Multivariate Cox regression analysis of MMD progression-free survival in patients with MCAD**

| **Variable** | **HR (95% CI)** | **P value** |
| --- | --- | --- |
| R4810K mutation | 8.42 (1.10–64.4) | **0.040** |
| Childhood onset (<17 yo) | 3.0 (0.915–9.56) | 0.071 |

**Table S2. Detailed analysis of stenosis progression patterns in Q hemispheres**

| **Q hemisphere, n=81** | ***RNF213* wild n=28** | **p.R4810K mutant n=53** |
| --- | --- | --- |
| Increase in stenosis degree, n(%) | 2 (7.1) | 8 (15.1) |
| Overall de novo stenosis in a new segment, n(%) | 1 (3.6) | 16 (30.2) |
| De novo stenosis in ICA, n(%) | 1 (3.6) | 14 (26.4) |
| ACA, n(%) | 0 | 2 (3.8) |
| MCA, n(%) | 0 | 0 (0) |
| PCA, n(%) | 0 | 0 (0) |
| BA, n(%) | 0 | 0 (0) |

**Table S3. Genotype–phenotype correlation of *RNF213* p.R4810K in unaffected hemisphere**

| **Unaffected hemisphere**  **n=38** | ***RNF213* wild n=17** | **p.R4810K mutant n=21** | **P value** |
| --- | --- | --- | --- |
| Follow-up, mo (IQR) | 64 (49–131) | 71 (44–165) | 0.750 |
| Progression to moyamoya hemisphere, n (%) | 0 (0) | 2 (9.5) | 0.492 |
| Progression of stenosis, n (%) | 1 (5.9) | 6 (28.6) | 0.104 |
| Hemispheres with revascularization surgery, n (%) | 0 (0) | 1 (4.8) | 1 |
| Hemispheres with cerebral infarction or hemorrhage during follow-up period, n (%) | 1 (5.9) | 1 (4.8) | 1 |
